# The Impact of Semimetallic Bismuth Electrical Contacts on Optical Properties of a MoS_2_ Monolayer

**DOI:** 10.1021/acsomega.6c06387

**Published:** 2026-10-18

**Authors:** Agata Zielińska, Mateusz Dyksik, Alessandro Surrente, Jonathan Eroms, Dieter Weiss, Paulina Płochocka, Mariusz Ciorga

**Affiliations:** † Department of Experimental Physics, 414290 Wrocław University of Science and Technology, Wybrzeże Wyspiańskiego 27, Wrocław 50-370, Poland; ‡ Institute for Experimental and Applied Physics, 9147 University of Regensburg, Universitätsstraße 31, Regensburg 93053, Germany; § Laboratoire National des Champs Magnétiques Intenses, 143 Avenue de Rangueil, Tolouse 31400, France

## Abstract

Achieving high performance in transition-metal dichalcogenide-based optoelectronic devices is challenging because the realization of an efficient electrical contacting scheme should not be obtained at the expense of their optical quality. Here, we present the results of optical studies of MoS_2_ monolayers that have been electrically contacted with bismuth and gold. The photoluminescence (PL) spectra of the samples contacted with both materials are significantly broadened. In the case of the bismuth-contacted sample, we observe an additional low-energy band in the PL spectrum, which we attribute to a defect state formed during the evaporation of Bi. Comparison of the intensities of the excitonic peak and the defect-related peak reveals that there is a correlation between the type of contacts and the optical properties of the contacted material.

## Graphical abstract

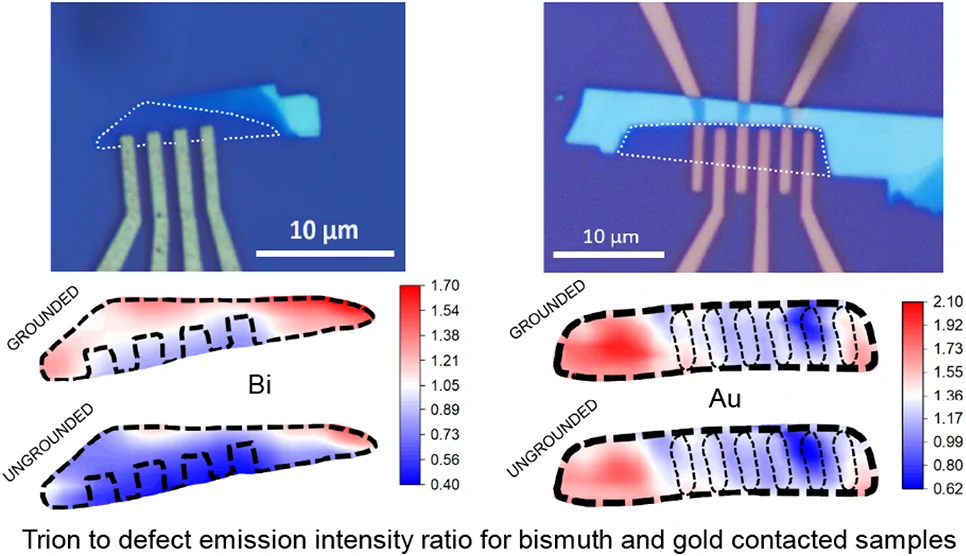

## Introduction

The unique electronic and optical properties of semiconducting van der Waals (vdW) materials make them both an attractive platform for fundamental studies of novel quantum effects (e.g., spin and valley Hall effects),^1–4^ proximity effects,^5,6^ as well as promising candidates for electronic and optoelectronic devices (e.g., field-effect transistors, photodetectors).^7–10^ Among the most extensively studied two-dimensional (2D) materials are the transition-metal dichalcogenides (TMDCs), in particular MoS_2_, MoSe_2_, WS_2_, and WSe_2_.^11–13^ These materials exhibit strongly bound excitons with high oscillator strength,^14–16^ which naturally led to intensive studies using various optical spectroscopic techniques and to optoelectronic applications.^17–20^

An efficient contact scheme is essential to fully exploit the electronic and optoelectronic potential of these materials, and this has been proven to be challenging in the case of 2D TMDCs. First of all, the 2D nature of these semiconductors makes interface effects much more important than those in the case of conventional bulk semiconductors. Standard ways of decreasing the contact resistance between a metal and a bulk semiconductor, involving substitutional doping at the contact region, are not applicable for 2D materials, as doping of thin layers is not optimized and generally could strongly modify their properties. These problems and different strategies developed to avoid them were thoroughly described in recent review papers.^21–23^ The application of these strategies has led to great progress in the realization of efficient electric contacts to TMDCs, either by optimizing metal/TMDC interfaces or by introducing graphene as a contact material,^24,25^ allowing the demonstration of scaled-down TMDC-based devices.^19,26–29^ However, there is still room to lower the contact resistance and move toward its quantum limit,^22^ which would be important not only for the scalability of the devices but also for the possibility of quantum transport studies at low temperatures.^30^

Recent reports demonstrate that very good low-resistive ohmic contacts to TMDCs can be achieved using a semimetal (bismuth or antimony) as a contact material.^31–40^ Due to the near-zero density of states at the Fermi level of the semimetal, the formation of gap states in TMDCs, which are responsible for the Fermi-level pinning at the metal/TMDC interface, is sufficiently suppressed, making it possible to obtain very low-resistance ohmic contacts. It has been demonstrated that these types of contacts should indeed allow one to reach the quantum limit in the contact resistance.^35^ Very recently, a very short transfer length of 2 nm was reported for Bi contacts to MoS_2_,^40^ suggesting that very good parameters of Bi contacts should indeed be preserved to very short contact lengths. Such contacts were also shown to work even at very low temperatures, which is very important for quantum transport investigations.^36,37^ To fully exploit the potential of semimetal contacts in TMDCs and their impact on optical properties of the contacted material, the exact physics at a semimetal/TMDC interface must be explored and understood in ways similar to those investigated for metal/TMDC interfaces, including theoretical simulations^41^ and experimental studies, taking into account such effects as charge transfer through contacts.^42–45^ However, combining optical and electrical investigations on the same sample is very challenging, due to the numerous processing steps required for efficient contacts, which can lead to degradation of the optical quality of the sample.^46^

In this work, our objective is to investigate the potential influence of bismuth contacts on the optical properties of a MoS_2_ monolayer, monitoring the emission response of MoS_2_ when contacted by Bi. With this goal in mind, we prepared a series of four monolayer samples of MoS_2_ with bismuth as the contact material. For all investigated samples, we measured the electrical performance, and two of the MoS_2_ flakes were widely optically investigated. Additionally, as a reference, we prepared and measured noncontacted and Au-contacted MoS_2_ flakes. Here, we present the results for 3 different samples, which we call A, B, and bare MoS_2_. Sample A is a MoS_2_ monolayer transferred on the Si/SiO_2_ substrate with bismuth contacts evaporated on the top of the flake. Sample B is a MoS_2_ monolayer transferred to previously evaporated gold contacts. Bare MoS_2_ is a MoS_2_ monolayer transferred on a Si/SiO_2_ substrate without any electrical contacts.

## Results and Discussion

We first discuss the optoelectronic properties of the representative sample of the back-gated MoS_2_ monolayers with bismuth contacts, which we will refer to as sample A. We show its micrograph in Figure 1a. The layer was placed on a Si/SiO_2_ substrate, and bismuth contacts were evaporated in an ultrahigh vacuum (UHV) system using an electron gun. We also prepared a series of reference samples with Au as the contact material. The details of the fabrication process are described in the Experimental Section.

**Figure 1:**
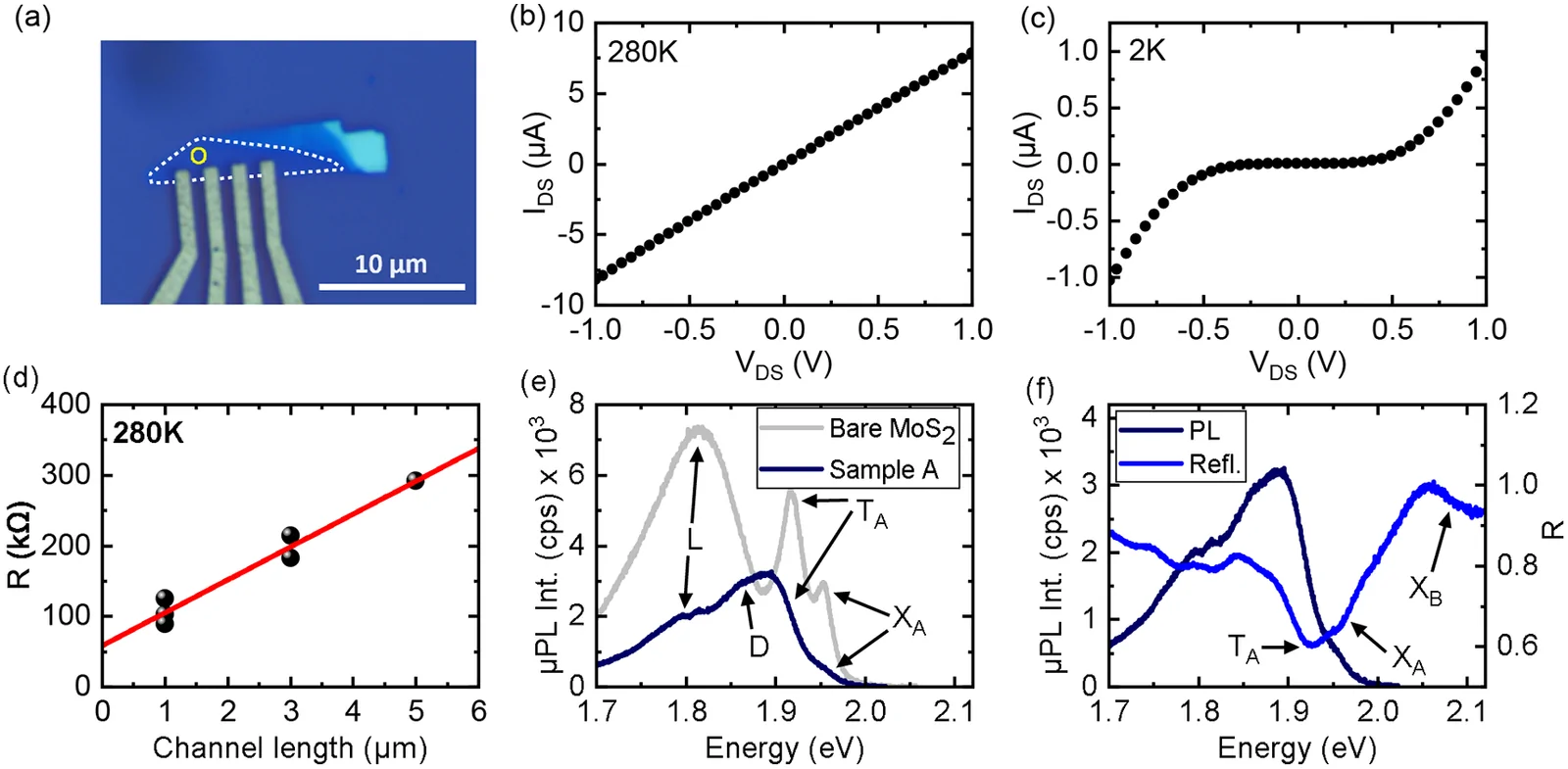
(a) Optical micrograph of sample A. All contacts are 1 μm wide and 118 nm high, with 1 μm spacing between them. MoS_2_ monolayer (marked with the white dotted line) is placed below the contacts. The yellow circle marks the spot where the PL spectrum presented in Figure 1­(e) was taken (spot size of ∼1.5 μm). (b) I–V curve for sample A for zero backgate voltage at 280 K. The contacts show clear ohmic behavior. (c) I–V curve for sample A at 2 K. The ohmic behavior is lost, but some conduction through the sample can still be observed. (d) Measured total resistance as a function of MoS_2_ channel length at 280 K for sample A. All data points are from the same sample, shown in (a), and are results of measurements between different pairs of contacts. Given that there are 4 contacts, there are 3 channels with a 1 μm length, 2 channels with 3 μm length and one channel with a 5 μm length. The total resistance increases linearly with the channel length. (e) μPL spectrum of sample A at 4.5 K (dark blue), taken from the spot marked with the yellow dot in Figure 1­(a), compared to a representative spectrum of the bare MoS_2_ flake (gray). Defect-related emission (L), new emission peak (D), trion emission (T_A_) and exciton emission (X_A_) are marked with arrows. (f) μPL (dark blue) and reflectivity (blue) spectra. The values of the reflectivity spectrum are arbitrary and relative to references taken from the silicon substrate. In the reflectivity spectrum, two transitions are well resolved: ∼1.923 eV (corresponding to T_A_) and ∼2.085 eV (corresponding to X_B_). The transition at ∼1.956 eV, corresponding to X_A_, is weakly visible.

### Electrical Measurements

We measured the current–voltage (I–V) curves of sample A for temperatures from 2 to 280 K. Figure 1b shows a representative I–V curve for zero back-gate voltage at 280 K. The curve shows ohmic behavior, consistent with previous reports,^31,35,37^ which is preserved down to 80 K. Contacts lose their Ohmic characteristics at temperatures below (see Figure 1c), but they still conduct current. Figure 1d presents the total resistance as a function of the channel length (distance between contacts). We can observe the resistance increasing linearly with the length of the channel, which indicates that the total resistance is dominated by the resistance of the channel.

### Optical Measurements

Sample A was optically investigated by measuring microreflectivity and microphotoluminescence (μPL) spectra at 4.5 K. Figure 1e shows a representative PL spectrum of sample A (dark blue line) taken from the middle of the flake (yellow circle in Figure 1a) with a spot diameter of ∼1.5 μm, and of the bare MoS_2_ monolayer exfoliated directly on the SiO_2_ substrate (gray line). The bare flake exhibits the typical low-temperature PL spectrum of MoS_2_, with three distinct emission lines related to various excitonic transitions. We attribute the high-energy peak at ∼1.95 eV to the recombination of the neutral exciton (X_A_), the peak at ∼1.92 eV to the recombination of the charged exciton (T_A_), while the low-energy broad band, labeled L in Figure 1e, is related to the recombination of excitons bound to defects present in the MoS_2_ monolayer.^17,47^ The higher intensity of the T_A_ line compared to that of the X_A_ line indicates charge doping, which, in the case of MoS_2_ has n-type character, as MoS_2_ is a naturally n-doped material.^48^ This is consistent with the observation of n-type current in electrical measurements.

The PL spectrum of sample A is significantly different from that of the bare flake. The total intensity of the observed emission is strongly quenched, and the emission lines are not so well resolved anymore. The maximum of the emission is now at ∼1.9 eV, i.e., at a slightly lower energy than the T_A_ line. This apparent redshift of the total emission is the result of a new, broad emission line, labeled D, appearing at ∼1.86 eV, i.e., between the T_A_ and L lines. To properly assign the observed emission lines to the corresponding optical transitions, we performed reflectivity measurements on the investigated samples. In Figure 1f we compare the PL spectrum of sample A with a reflectivity spectrum measured on another MoS_2_ monolayer sample with bismuth contacts. The transitions clearly visible in the reflectivity spectrum correspond to the charged exciton at 1.92 eV, the A exciton at 1.96 eV, and the B exciton at 2.1 eV.^11^ The presence of a trion-related resonance in the reflectivity spectrum suggests significant doping, which induces a partial transfer of oscillator strength from the exciton to the charged exciton transition.^49,50^ All exciton transitions in reflectivity are in good agreement with literature values.^51^ The lack of a clear red shift in the excitonic transitions indicates a negligible effect of strain, which could potentially accumulate in the sample following the contact evaporation process. However, there is no visible transition in the reflectivity at ∼1.86 eV, which suggests that line D corresponds to defect-related emission—probably an exciton localized on defects. To further investigate the D line, we measured the dependence of PL power in the range of 1 μW to 500 μW and fitted the obtained spectra with a multiple Gauss function. All peaks showed approximately linear dependence, indicating the excitonic origin of all peaks. The results of the power dependence measurements are presented in the Supporting Information (Figures S1 and S2).

It is important to mention that a spectrum similar to that measured for sample A, with an additional emission line below the *T*_*A*_ line, was also observed for the other optically investigated Bi-contacted flake (see Figure S3 in the Supporting Information).

### Reference Sample with Gold Contacts

To check whether the D line is characteristic only for the Bi-contacted samples, we also prepared and measured a set of samples with Au contacts,^52^ and an optical microscope image of a representative Au-contacted sample (sample B) is presented in Figure 2a. Whereas more details on the fabrication, processing, and results of optical and electrical measurements of these samples can be found in the Supporting Information and in ref 52. Here we present the most relevant features. In Figure 2b and c, we show, respectively, representative PL and reflectance spectra of sample B at 4.5 K. Similar to sample A, the PL lines are broadened compared to those of bare MoS_2_, but there is no signature of any additional line close to the T_A_ line. Similar PL spectra were obtained for other Au-contacted samples.

**Figure 2:**
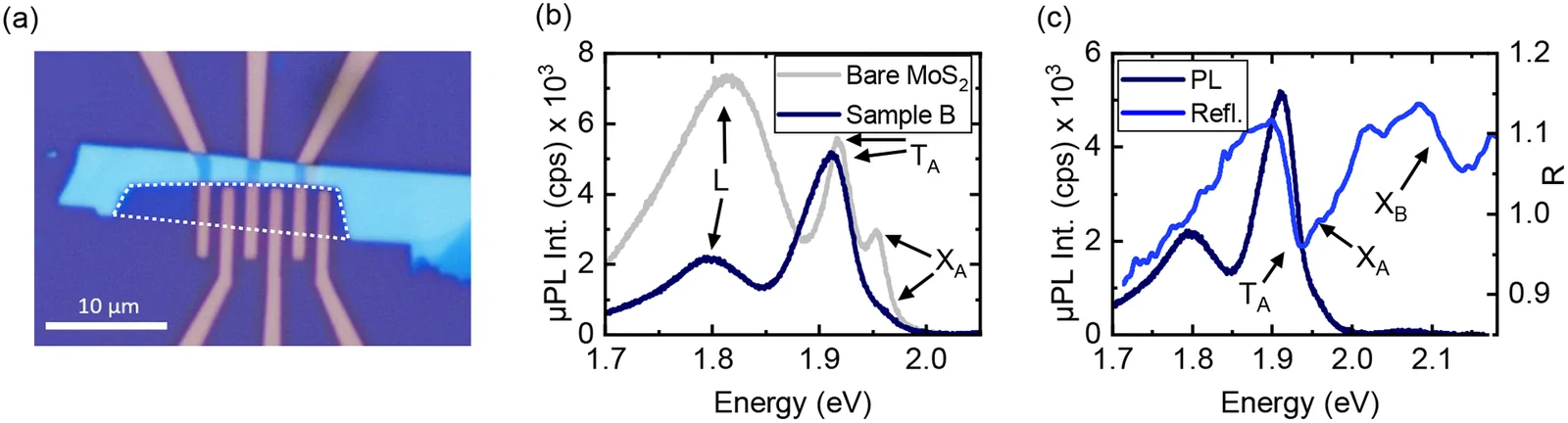
(a) Optical microscope image of sample B. All contacts are 25 nm high. The MoS_2_ monolayer (marked with the white dotted line) is placed on top of the contacts. Adapted from ref 52. (b) μPL spectrum of sample B (dark blue) compared to the μPL spectrum of the bare MoS_2_ flake (gray) measured at 4.5 K. Defect-related emission (L), trion emission (T_A_) and exciton emission (X_A_) are marked with arrows. (c) μPL (dark blue) and reflectivity (blue) spectra of sample B. The values of the reflectivity spectrum are arbitrary and relative to references taken from the silicon substrate. In the reflectivity spectrum, two transitions are well resolved: ∼1.92 eV and ∼2.1 eV. The transition at ∼1.95 eV is weakly visible.

The observation of line D in the PL spectra of both measured Bi-contacted samples and the lack thereof in the measured spectra of Au-contacted samples suggest that its presence might be a result of bringing the MoS_2_ flake into contact with Bi. This is consistent with the results of similar experiments performed by Feng et al.^53^ on CVD-grown MoS_2_ monolayers covered with thin semimetal layers deposited on MoS_2_ by molecular beam epitaxy, where the authors observed a red shift of the PL spectrum at room temperature. They explained it as a consequence of the transfer of electrons from Bi to MoS_2_, resulting in an increase in the electron concentration in MoS_2_ and an increase in the intensity of the emission of T_A_ relative to that of X_A_. This transfer of electrons from Bi to MoS_2_ is not unexpected, as theoretical calculations show that at the Bi/MoS_2_ interfaces, electrons tend to accumulate at the S atoms due to their stronger electronegativity than for the Bi atoms.^53,54^ However, because the charge-transfer length is very short-distance, a change in the PL shape would be expected mainly in the vicinity of the contacts. This is not what we observe, as the D line appears on the entire surface of the sample, also on parts of the flake far from the contacts, as shown in Figure 3. We believe that the possible diffusion of Bi atoms into MoS_2_, which would result in electron doping, could play a role here. However, there is not much information in the literature on doping of MoS_2_ thin films with Bi or on diffusion of Bi atoms in MoS_2_.^55^ Qumar et al.^56^ observed a small redshift of total PL emission from Bi-doped MoS_2_ nanosheets but did not explicitly state whether Bi-substituted S or Mo atoms. Rahman et al.^39^ reported on uniform Bi doping of the MoS_2_ monolayer as a result of evaporation of the Bi thin film, but did not present any low-temperature PL spectra that could be compared with our results. The possibility of diffusion of Bi atoms at Bi/MoS_2_ interfaces was discussed by Kim et al.^57^

**Figure 3:**
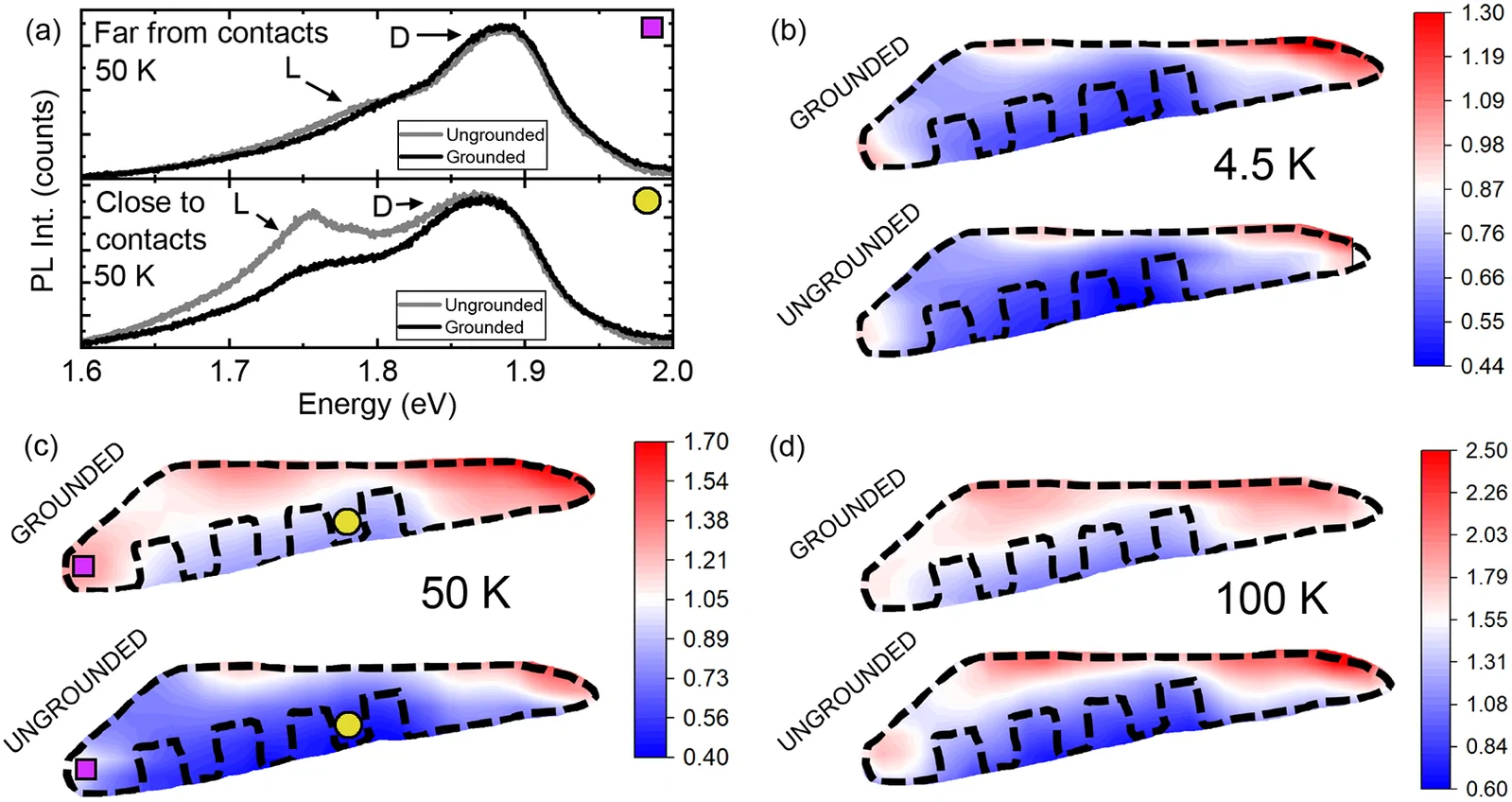
(a) Comparison of PL spectra of two different spots on sample A for contacts grounded and ungrounded at 50 K. The violet square corresponds to the spot far from the contacts, and the yellow circle corresponds to the spot close to the contacts (both spots are marked on the corresponding map below). (b)–(d) Trion-to-defect ratio for contacts grounded and ungrounded at 4.5 K, 50 K, and 100 K for sample A. The dashed lines represent the edges of the flake and the contour of the contacts. For 50 K a substantial difference is visible in the grounded contacts compared to the ungrounded contacts. In case of 4.5 K and 100 K we observe smaller differences in the map; however, the trion-to-defect ratio is also bigger for the grounded contacts.

In view of the above, we must also discuss the possible role of the evaporation process of bismuth since a certain impact of evaporation on the structure of the flake directly below the contacts could not be easily excluded. However, as discussed before, the lack of a clear red-shift in the excitonic transitions indicates that there is no strain in the sample, which could be created during evaporation. On the other hand, one could argue that it was the evaporation process that induced the diffusion of Bi atoms into the monolayer. Of course, our interpretation of the D line originating from the diffusion of Bi must be treated very speculatively. To verify the claim, more direct evidence would be necessary, involving compositional or structural measurements.^58^

### Effect of Grounding the Contacts on Optical Properties

To gain more insight on the origin of the D-line, we checked how the presence of excess free carriers in the flake affects the PL spectrum; namely, we compared μPL maps taken with all electrical contacts ungrounded with those taken with all contacts grounded, assuming that grounding removes excess charge carriers. We performed μPL measurements on sample A at T = 4.5 K, 50 K, 100 K, and 300 K, mapping out the whole sample. In Figure 3a we plot the PL spectra taken at two different spots on the sample—far from contacts (the violet square) and close to contacts (the yellow circle). Figure 3b–d shows μPL maps of the trion-to-defect ratio at selected temperatures for sample A, where the intensity ratio was obtained by integration in the ranges corresponding to the trion at ∼1.9 eV (0.14 eV range width) and to the defect-bound excitons at ∼1.8 eV (0.19 eV range width), where the former range includes the line D at 1.86 eV. The ranges were red-shifted with temperature, according to the temperature shift of the peaks. Generally, in the case of sample A, there is a substantial difference in the optical spectrum when we ground the contacts or leave them ungrounded. Grounding leads to a decrease in the intensity of defect L and to negligible changes in the intensity of trion and D lines, as illustrated in Figure 3a. We observe the quench of defect L emission by grounding on both spots, but in case of the spot close to the contacts, this effect is more pronounced. In maps shown in Figure 3b–d, one can see that the trion-to-defect ratio is clearly larger for grounded contacts, indicating that excess charge carriers transferred across the contacts bind mainly to the defects present in the crystal, while the trion and exciton emission lines are not significantly influenced. The effect is the strongest at 50 K. We relate this to the fact that at this temperature the contacts have a much smaller resistance compared to the resistance at 4.5 K, which increases the possibility of charge transfer. In addition, at low temperatures, the carriers are tightly bound, which means they can not be freely transferred through the contacts. However, the defect emission is generally significantly suppressed at higher temperatures, which would make comparison with the trion intensity impossible. We can conclude that the charge transfer through the working contacts mostly affects the excess charge in the flake. As D emission is not significantly influenced by grounding, it confirms our assumption that the D and L peaks have different origins. Although both are related to excitons bound to defects, different types of defects are involved in each case, with different binding energies of the corresponding excitons.

To analyze in more depth the effects described above, we measured the temperature dependence of the PL spectrum of sample A in the range of 5 to 300 K. We fitted the curves with four Gaussian peaks to obtain the emission intensity of the L and D lines as a function of temperature, and fitted the Arrhenius plot of the obtained data to extract the activation energies of the relevant processes of exciton dissociation.^59^ In the case of line L, we used the formula with two activation energies for PL quenching:

1 $$I=\frac{I_{0}}{1+A_{1}\exp\left(-E_{1}/k_{B}T\right)+A_{2}\exp(-E_{2}/k_{B}T)}$$

In case of peak D, we used the formula with three activation energies—one for the increase of the PL at low temperatures and two for PL quenching at higher temperatures:

2 $$I=\frac{I_{0}[1+\left(A_{1}\exp\left(-E_{1}/k_{B}T\right)\right]}{1+A_{2}\exp\left(-E_{2}/k_{B}T\right)+A_{3}\exp(-E_{3}/k_{B}T)}$$

where *E*_*i*_ is the activation energy and *A*_*i*_ is the amplitude of the process.

The results of the fitting are presented in Figure 4. The L line was fitted with a function containing two activation energies for PL quench, as only one activation energy was not enough to reproduce the experimental data. We obtain two activation energies of (4.6 ± 2.0) meV and (37 ± 14) meV. The D line was fitted with a function containing three activation energies—one for the increase of PL and two for PL quench. It was necessary to use three activation energies to correctly reproduce the data. We obtained 2.5 ± 0.8 meV activation energy for the PL increase, and 5.2 ± 0.5 meV and 46 ± 10 meV for the two processes of PL quench. We compared the obtained values with the binding energies of various excitonic complexes on different types of defects in transition-metal dichalcogenides, calculated with Monte Carlo simulations performed by Mostaani et al.^60^ The first activation energy for the L line could correspond to an exciton bound on donor (*D* ^+^ *X*) or acceptor (*A* ^–^ *X*) in the lattice structure. Their binding energies are equal to 7.2 and 2.7 meV, respectively. Taking into account the uncertainty of the obtained values, we cannot assign the first activation energy to any particular exciton complex. The second activation energy equal to 37 meV can correspond to a negative trion bound to a donor (*D* ^0^ *X*) or a positive trion bound to an acceptor (*A* ^0^ *X*). Their binding energies are equal to 32.4 and 31.7 meV, respectively. Taking into account the fact that MoS_2_ is naturally n-doped, *D* ^0^ *X* is more probable. In our sample, it could mean a negative trion bound to a hydrogenized sulfur vacancy.^61^ Two excitonic complexes emitting near 1.8 eV would be in agreement with a PL shape that is very broad and looks like more than one Gaussian. In the case of line D we observe an initial PL increase with an activation energy of 2.5 meV, which can correspond to *A* ^–^ *X* dissociation. It could mean that the carriers previously dissociated from line L are then bound to the states responsible for the line D emission state, increasing the PL intensity. The additional carriers can also come from a different reservoir, which is not described here. The first activation energy of the PL quench, 5.2 meV, may correspond to dissociation of *D* ^+^ *X* or *A* ^–^ *X*. The second activation energy equal to 46 meV may correspond to a biexciton bound to acceptor (*A* ^–^ *XX*), whose activation energy is equal to 48 meV. If we assume diffusion of Bi atoms during the evaporation process and substitution of sulfur atoms, then Bi atoms would serve as acceptors, as bismuth has one valence electron less than sulfur. Taking this into account, we can conclude that the first activation energy for PL quench probably corresponds to the dissociation of an exciton bound to a Bi atom acceptor (*A* ^–^ *X*) and the second activation energy corresponds to a biexciton bound to a Bi atom acceptor (*A* ^–^ *XX*). The fact that we do not observe free biexciton emission could mean that our sample structure has so many defects that all biexcitons are bound to them and there are no free biexcitons. The majority of excitons and trions could also be bound to defects and it would be consistent with the very low PL emission of the free complexes. In Supporting Information we also show the trion-to-defect maps of sample B at 50 K with grounded and ungrounded contacts. There is no significant difference between the two maps, which probably results from the high resistance of the Schottky contacts at low temperatures.

**Figure 4:**
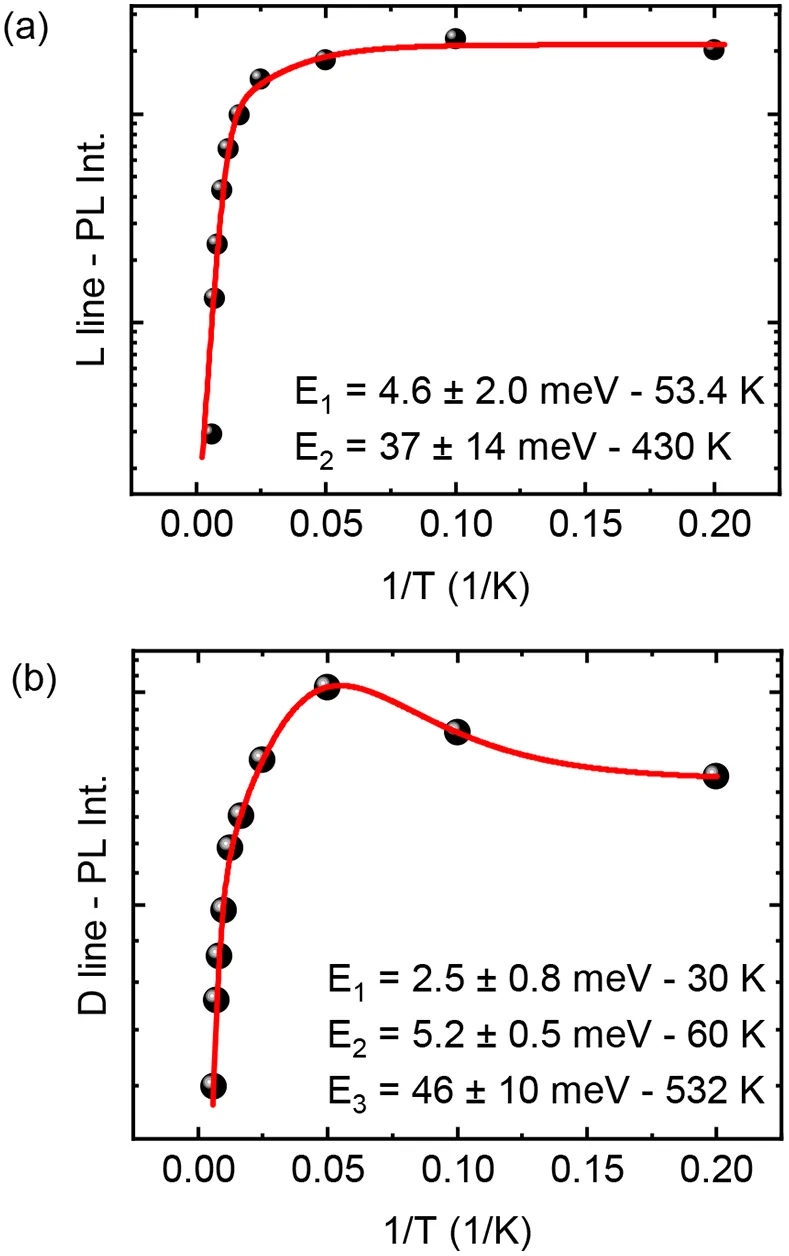
Arrhenius plot of peak L (a) and D (b) intensity with the Arrhenius equations fitted to the data.

## Conclusions

In conclusion, we present the results of investigations of the optical properties of MoS_2_ monolayers that were electrically contacted with semimetallic bismuth. The contacts show ohmic behavior in the temperature range from 280 to 80 K, whereas the gold contacts used as a reference exhibit Schottky characteristics throughout the entire range. The two materials used to fabricate the contacts also significantly affect the optical properties of the MoS_2_ monolayers, broadening the emission lines and decreasing the overall intensity. In the case of the bismuth contacts, an additional defect band in the PL spectrum appears, that might be attributed to the diffusion of bismuth atoms during the deposition process.

Finally, by comparing the PL spectra of grounded and nongrounded samples at 50 K, we demonstrate that grounded bismuth contacts significantly reduce the intensity of defect-bound excitons, which we attribute to removing the charges bound to defects via charge transfer through the grounded contacts. This effect is not observed in the sample with gold contacts, which is associated with its Schottky-like characteristics. Overall, our results confirm that bismuth contacts are indeed very strong candidates for electrical contacts in high-quality TMD-based optoelectronic devices. However, their effect on the optical spectrum of the TMDC requires further exploration.

## Experimental Section

### Fabrication of Electrical Contacts

Silicon substrates with a 285 nm SiO_2_ layer on top were first cleaned in oxygen plasma for 5 min. Then, the substrates were spin-coated twice with poly­(methyl methacrylate) (PMMA) layers (200k 9% and 950k 5%) and baked for 5 min at 150 ^◦^C after each process. The contacts pattern was written in a Zeiss Auriga SEM on each substrate by the electron-beam lithography using a self-prepared mask. The pattern was then developed in methyl isobutyl ketone (MIBK) diluted in isopropanol with 1:3 ratio and cleaned in isopropanol. Afterward, the substrates were put in an UHV system where 5 nm of titanium and 20 nm of gold or 38 nm of bismuth and 80 nm of gold were evaporated. In case of the bismuth contacts, the evaporation rate was ∼ 0.6 Å/s for bismuth and ∼1.1 Å/s for gold. The evaporation was carried out at a pressure of ∼1 × 10^–7^ mbar. Finally, the PMMA resist was dissolved in acetone heated to 60 ^◦^C.

### Exfoliation and Transfer

The MoS_2_ flakes were fabricated from bulk crystals (HQ Graphene) by mechanical exfoliation and transferred to polydimethylsiloxane (PDMS) gel films. The monolayers were found in an optical microscope, taking advantage of the thickness-dependent optical contrast. Finally, the monolayers were dry-transferred onto substrates heated to 80 ^◦^C.

### AFM Ironing

The parts of the MoS_2_ flake laying on each of the gold contacts were one-way-scanned by a Park AFM in contact mode with a non-contact cantilever in order to improve the quality and cleanness of the fabricated heterostructures. In the following, we refer to this process as AFM ironing.^62–64^ The ironing force was set to 100 nN, and the scan rate was set to 0.6 Hz. That results in a tip velocity of ∼5 μm/s. The resolution was set to 512 px, which gives 1 to 2 nm between each line scan.

### Transport Measurements

I–V characteristics were measured in a wide temperature range in a superconducting magnet system (Oxford Instruments). A Yokogawa 7651 was used as the voltage source, and an Agilent 34410A multimeter with an Ithaco 1211 preamplifier was used to measure the drain-source voltage. A Keithley SMU 2400 source meter was used to set backgate voltages.

### Optical Spectroscopy

Measurements were performed in an optical helium-flow cryostat (Oxford Instruments). The excitation source for microphotoluminescence was a 532 nm continuous-wave laser. The white-light source for microreflectance was a tungsten-halogen light source (Thorlabs), which was spatially filtered through a 50 μm pinhole, resulting in a final spot size of ∼4 μm. Laser light and white light were focused on the sample surface with 0.75 NA, and a 50× microscope objective (Mitutoyo). The same objective was used to collect the output signal, which was then directed to a spectrometer (500 mm focal length, 300 groove/mm grating, and 150 μm slit width) equipped with a liquid-nitrogen-cooled CCD camera (Princeton Instruments).

## Supplementary Material



## Notes

Power dependence of PL emission from sample A; PL of the second sample with Bi contacts; temperature series of PL emission from sample A; trion-to-defect emission intensity ratio of sample B; and PL map of sample B (PDF)
