# Supplementary material for: The Impact of Semimetallic Bismuth Electrical Contacts on Optical Properties of a MoS2 Monolayer
Source: ACS Omega. 2026 Oct 18;11(43):66992–9. doi: 10.1021/acsomega.6c06387 (PMC13737922; doi:10.1021/acsomega.6c06387)
Supplement: Supplementary file 1 [file ao6c06387_si_001.pdf]

Supporting Information for: The impact of semimetallic bismuth electrical contacts on optical properties of a MoS_2 monolayer

Agata Zielińska*[1], Mateusz Dyksik[1], Alessandro Surrente[1], Jonathan Eroms[2], Dieter Weiss[2], Paulina Płochocka[1,3], and Mariusz Ciorga[1]

[1]Department of Experimental Physics, Wrocław University of Science and Technology, Wybrzeże Wyspiańskiego 27, 50-370, Wrocław, Poland
[2]Institute for Experimental and Applied Physics, University of Regensburg, Universitätsstraße 31, 93053, Regensburg, Germany
[3]Laboratoire National des Champs Magnétiques Intenses, 143 avenue de Rangueil, 31400, Tolouse, France

*Email: agata.zielinska@pwr.edu.pl

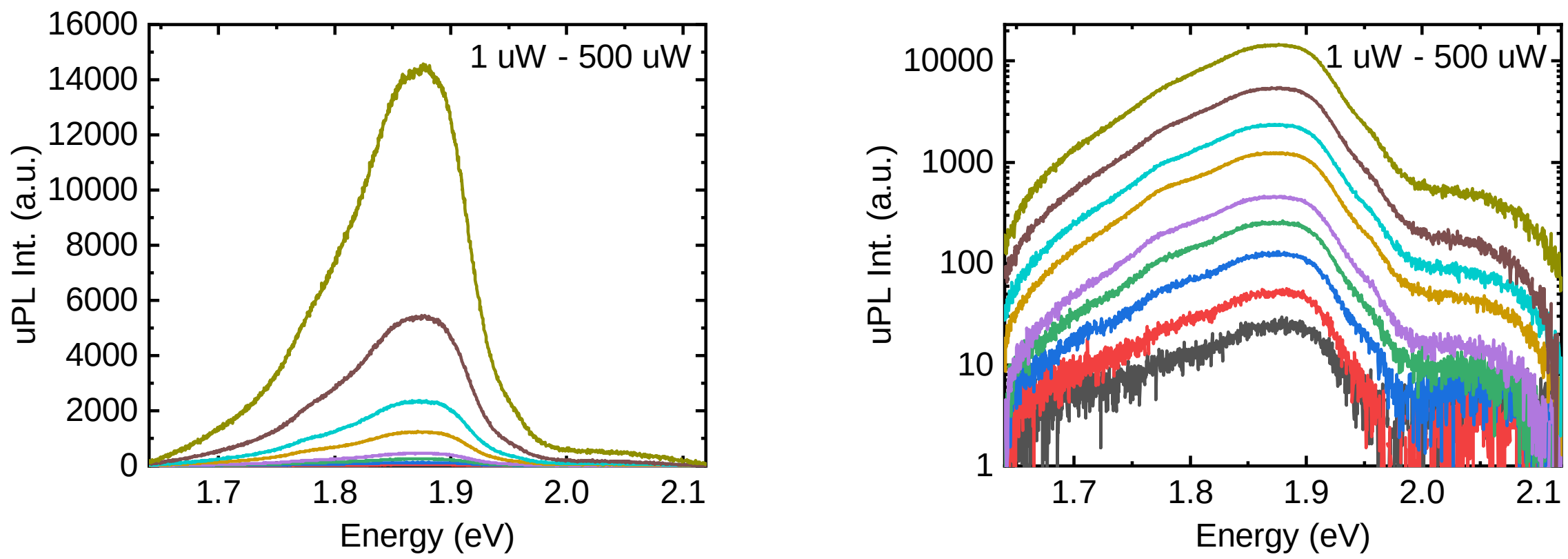

Figure 1: Power dependence of PL emission from sample A. PL was measured in range of 1 µW – 500 µW, with the excitation wavelength of 532 nm (2.33 eV).

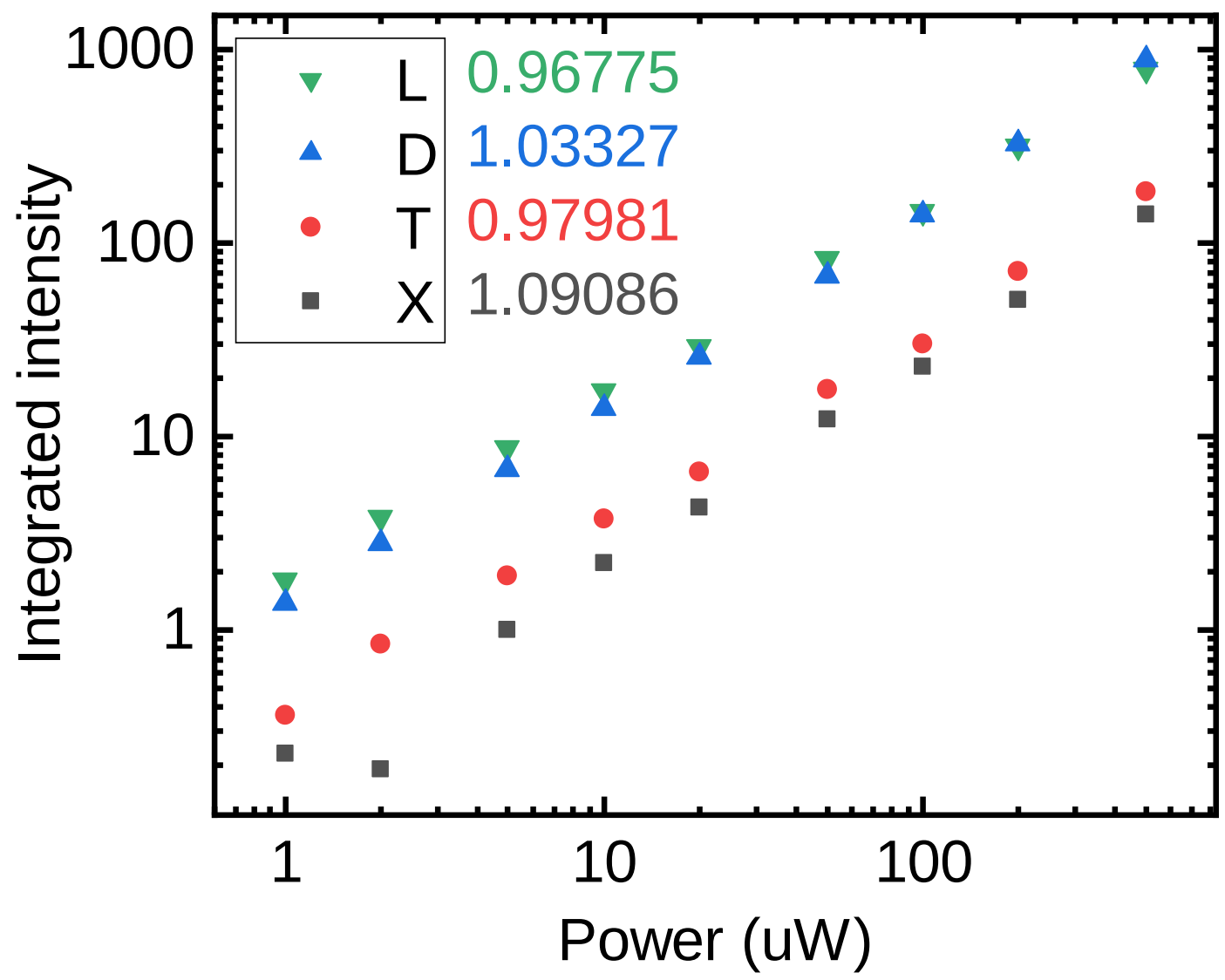

Figure 2: Integrated intensity emission for all the peaks in the PL of sample A. The spectra were fitted with a multiple Gauss function using the least squares method. After fitting the PL for the highest laser power, the peaks energies were fixed to reduce the number of fitting parameters for the rest of the spectra, as the peak energies are not expected to change in the used power range. The slopes obtained from the linear fits for all peaks are written on the graph in color. All peaks show nearly linear power dependence (slope ~ 1).

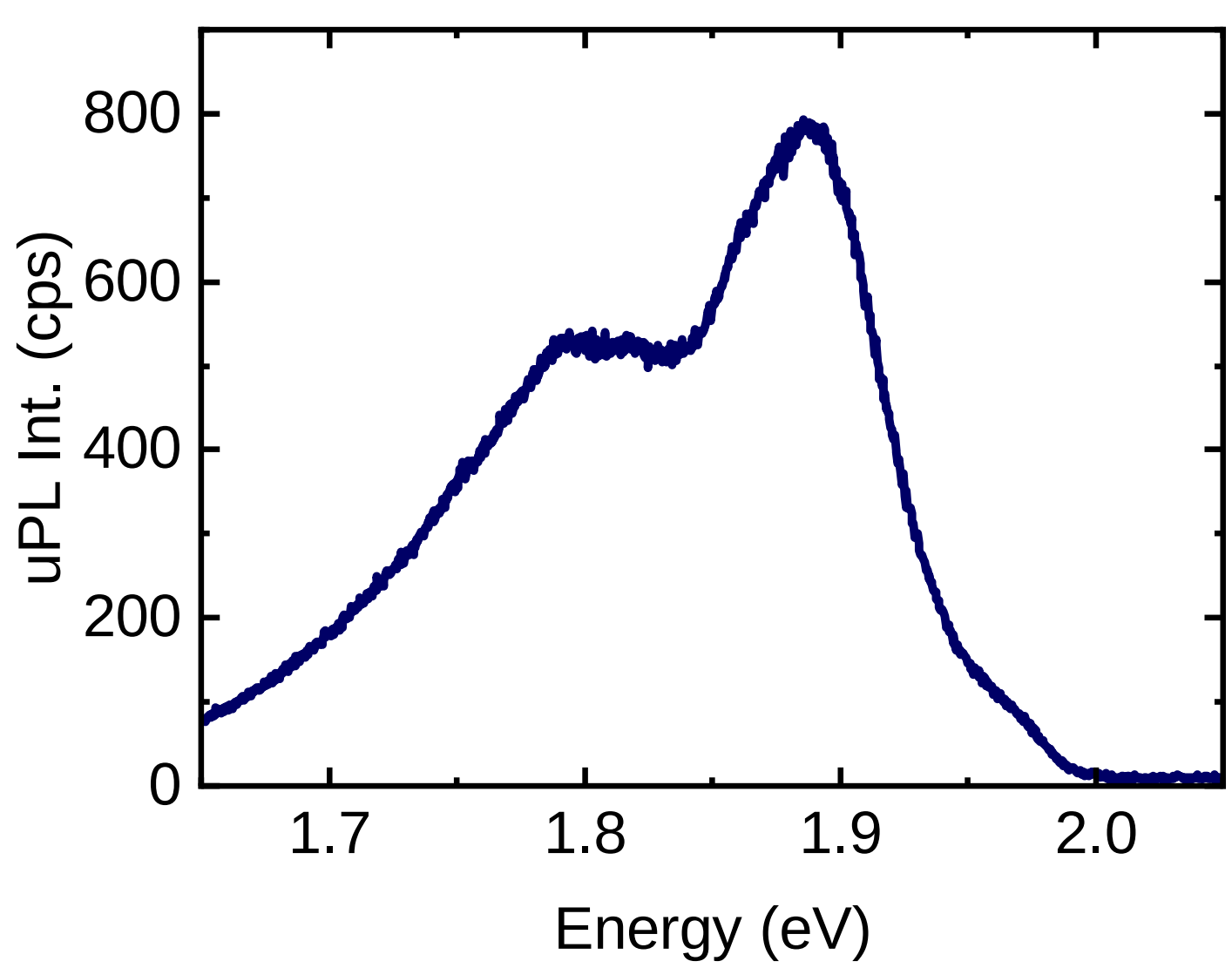

Figure 3: µPL of the second sample with Bi contacts. The emission peak at $\sim 1.86\,\mathrm{eV}$ is visible and the maximum of the whole PL spectrum is below $1.9\,\mathrm{eV}$.

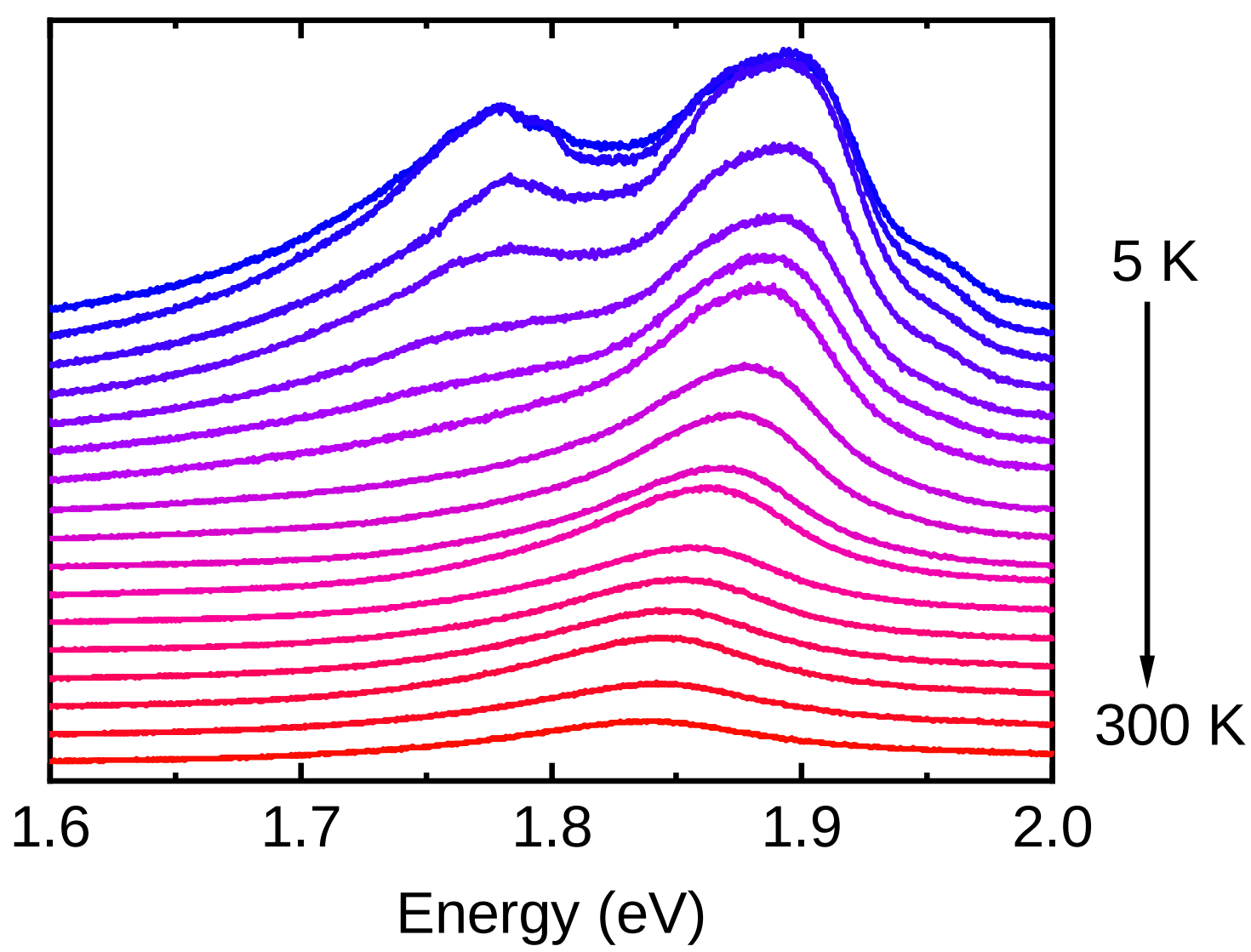

Figure 4: Temperature series of sample A measured in range 5–300 K with a 20 K step. We can observe that the defect D is suppressed with increasing the temperature, but with a much more slower rate than the L line.

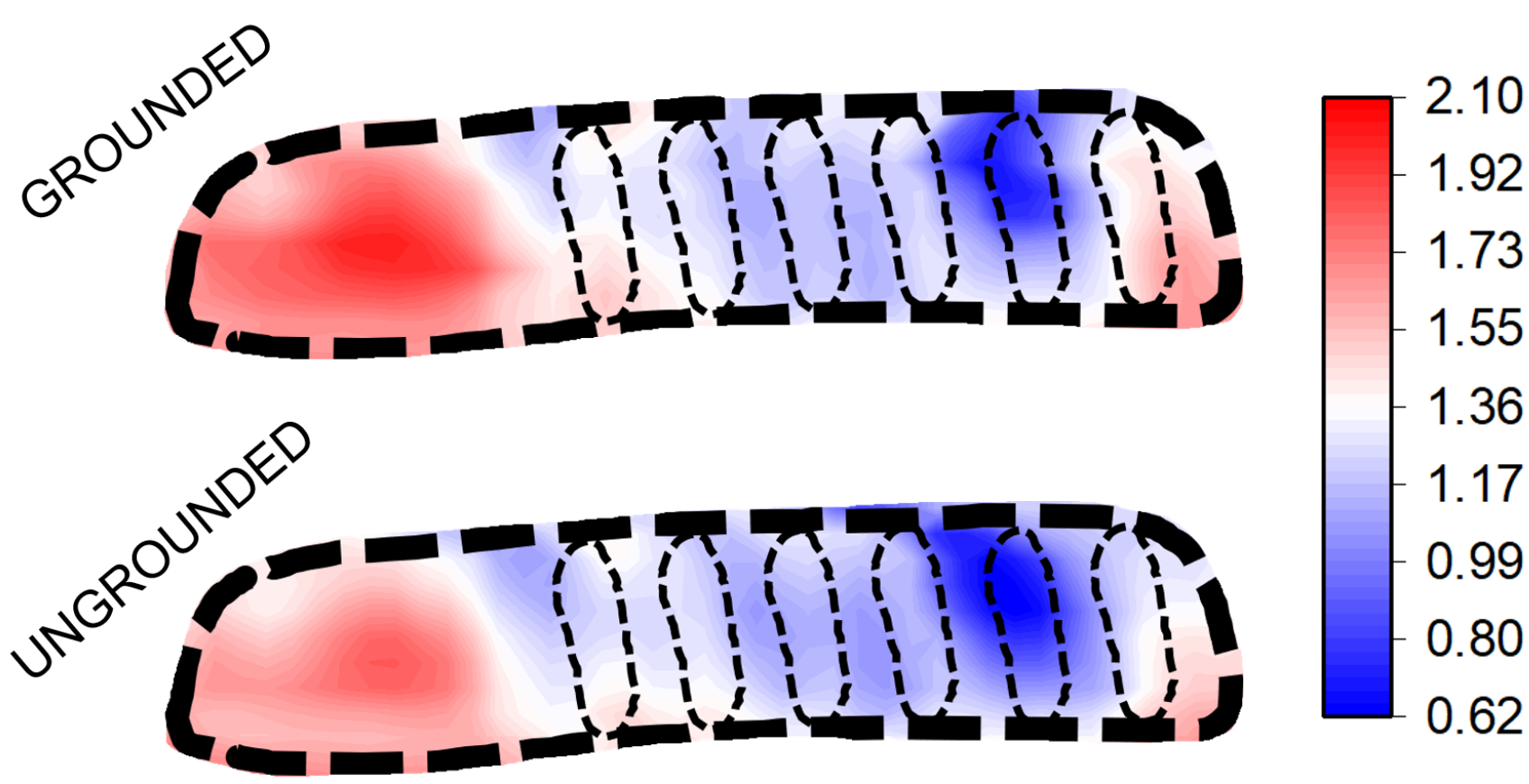

Figure 5: Trion to defect ratio of sample B at 50 K. The differences between grounded and ungrounded contacts at both temperatures are much less visible than for sample A.

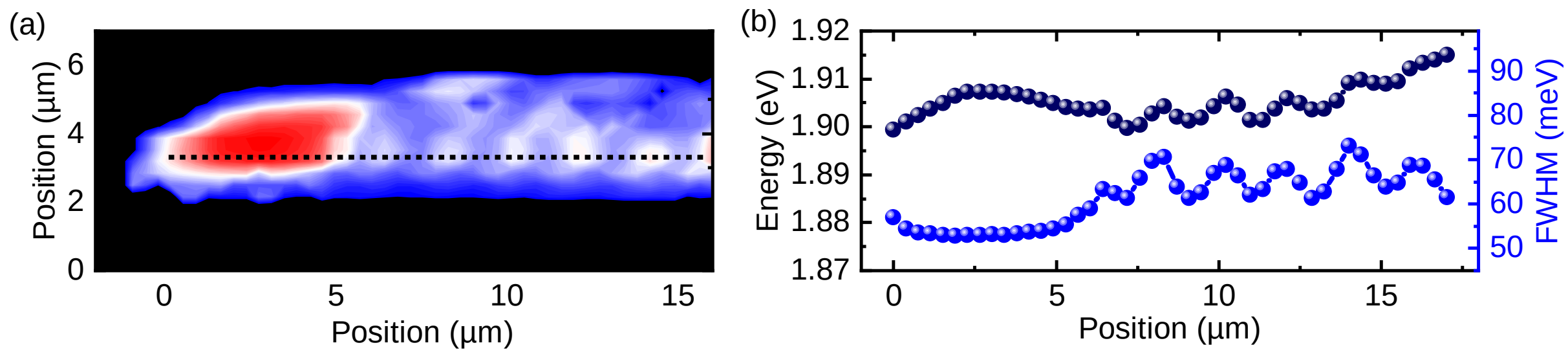

Figure 6: (a) μPL map obtained by integrating the spectral range corresponding to the trion and neutral exciton emission (1.86–2 eV). The bright parts in the middle of the map denote the positions of the contacts. (b) Position dependence of the energy centroid (center of mass) and the full width at half maximum (FWHM) of the integrated peak extracted across the dashed line in (a). Parts of the flake laying on the contacts characterize with higher intensity, smaller centroid and smaller FWHM of the integrated emission. The emission from the left part of the flake (far away from the contacts) is characterized by much smaller broadening than the parts on or between contacts. It means that the emission from the flake around contacts is inhomogeneous, which is probably also a consequence of the strain induced during transfer of the flake on the contacts. The surface plasmon polaritons may also play a role in the PL shape by changing its intensity and peak energies while illuminating parts of the layer laying on the gold contacts. The intensity and the emission energy changing across the dashed line in (a) are probably a combination of these two effects.
